# The phylogeny of fossil whip spiders

**DOI:** 10.1186/s12862-017-0931-1

**Published:** 2017-04-21

**Authors:** Russell J. Garwood, Jason A. Dunlop, Brian J. Knecht, Thomas A. Hegna

**Affiliations:** 1 School of Earth and Environmental Sciences, The University of Manchester, Manchester, M13 9PL UK; 2 Department of Earth Sciences, The Natural History Museum, Cromwell Road, London, SW7 5BD UK; 3 Museum für Naturkunde, Leibniz Institute for Research on Evolution and Biodiversity, Invalidenstraße 43, D-10115 Berlin, Germany; 4 Department of Geology, Western Illinois University, Tillman Hall 113, 1 University Circle, Macomb, IL 61455 USA

**Keywords:** Amblypygi, Coal Measures, Amber, Fossil, Systematics, Pennsylvanian

## Abstract

**Background:**

Arachnids are a highly successful group of land-dwelling arthropods. They are major contributors to modern terrestrial ecosystems, and have a deep evolutionary history. Whip spiders (Arachnida, Amblypygi), are one of the smaller arachnid orders with ca. 190 living species. Here we restudy one of the oldest fossil representatives of the group, *Graeophonus anglicus* Pocock, 1911 from the Late Carboniferous (Duckmantian, ca. 315 Ma) British Middle Coal Measures of the West Midlands, UK. Using X-ray microtomography, our principal aim was to resolve details of the limbs and mouthparts which would allow us to test whether this fossil belongs in the extant, relict family Paracharontidae; represented today by a single, blind species *Paracharon caecus* Hansen, 1921.

**Results:**

Tomography reveals several novel and significant character states for *G. anglicus*; most notably in the chelicerae, pedipalps and walking legs. These allowed it to be scored into a phylogenetic analysis together with the recently described *Paracharonopsis cambayensis* Engel & Grimaldi, 2014 from the Eocene (ca. 52 Ma) Cambay amber, and *Kronocharon prendinii* Engel & Grimaldi, 2014 from Cretaceous (ca. 99 Ma) Burmese amber. We recovered relationships of the form ((*Graeophonus* (*Paracharonopsis* + *Paracharon*)) + (*Charinus* (*Stygophrynus* (*Kronocharon* (*Charon* (*Musicodamon* + *Paraphrynus*)))))). This tree largely reflects Peter Weygoldt’s 1996 classification with its basic split into Paleoamblypygi and Euamblypygi lineages; we were able to score several of his characters for the first time in fossils. Our analysis draws into question the monophyly of the family Charontidae.

**Conclusions:**

Our data suggest that *Graeophonus* is a crown group amblypygid, and falls within a monophyletic Paleoamblypgi clade, but outside the family Paracharontidae (= *Paracharonopsis* + *Paracharon*). Our results also suggest a new placement for the Burmese amber genus *Kronocharon*, a node further down from its original position. Overall, we offer a broad phylogenetic framework for both the fossil and Recent whip spiders against which future discoveries can be tested.

**Electronic supplementary material:**

The online version of this article (doi:10.1186/s12862-017-0931-1) contains supplementary material, which is available to authorized users.

## Background

**Fig. 1:**
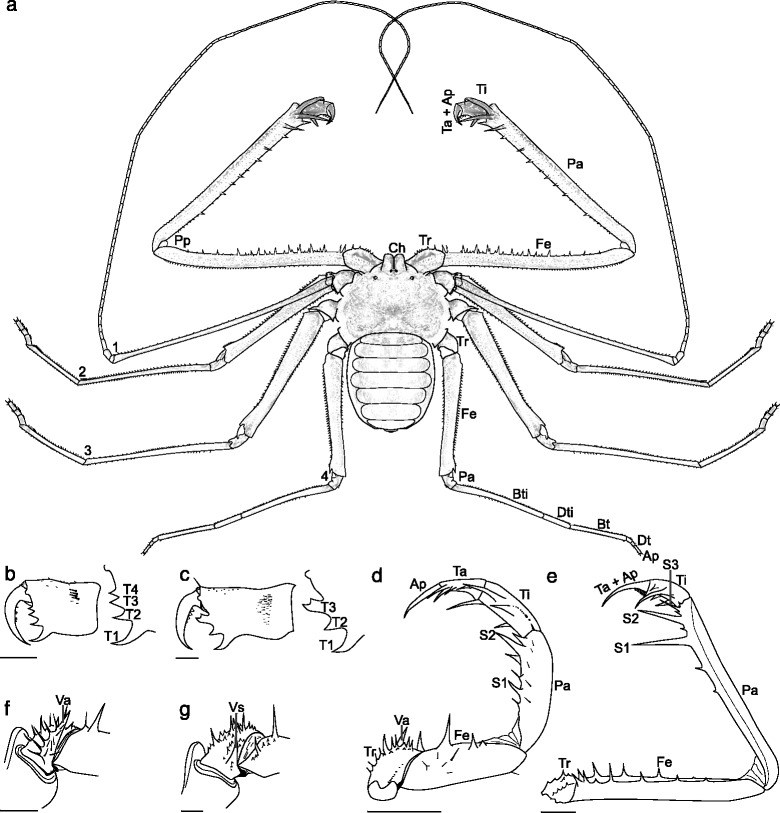
Guide to the morphology of Amblypygids, and terminology used herein. **a** Gross morphology of the extant amblypygid *Damon* sp.—a species typically 24–35 mm in length when fully grown—as seen in dorsal view; drawn from an unnumbered specimen in the Museum für Naturkunde, Berlin. **b** The chelicera of *Paracharon caecus* in lateral view, a species with four cheliceral teeth; scale bar equals 0.5 mm (redrawn from ref [34], Figure 1). **c** The chelicera of *Charon grayi* with three cheliceral teeth, the distalmost bicuspate; scale bar equals 1 mm (redrawn from ref [34], Figure 3). **d** *Paracharon caecus* palp in dorsal view, with only two patellar spines; scale bar equals 1 mm (redrawn from ref [34], Figure 12). **e** Pedipalp of *Charon grayi* which has three palpal spines forming a catching basket; scale bar equals 2 mm (redrawn from ref [34], Figure 15). **f** Ventral left trochanter of *Charinus montanus* palp, showing a prominent anterior ventral apophysis; scale bar equals 0.5 mm (redrawn from ref [34], Figure 7). **g** *Damon variegatus* ventral left palp trochanter, ventral anterior apophysis reduced to a spine; scale bar equals 0.5 mm (redrawn from ref [34], Figure 8). Abbreviations: 1–4—legs 1–4; Ap—apotele; Bt—basitarsus; Bti—basitibia; Ch—chelicerae; Dt—distitarsus; Dti—distitibia; Fe—femur; Pa—patella; Pp—pedipalps; S1-3—spines 1–3, numbers proximally to distally; T1-4—cheliceral teeth 1–4 numbered from ventral to dorsal; Ta—tarsus; Ti—tibia; Tr—trochanter; Va—palpal ventral anterior apophysis; Vs—palpal ventral anterior spine

Whip spiders (Arachnida: Amblypygi) are distinctive creatures (Fig. 1) with a long, slender, antenniform first pair of legs. These whip-like appendages give the group its name, although they are occasionally referred to as tailless whip scorpions because they also resemble a related group of arachnids, the whip scorpions (Uropygi), albeit without the whip scorpion’s flagelliform telson. Both whip spiders and whip scorpions belong—together with spiders (Araneae) and schizomids (Schizomida)—to the arachnid clade Tetrapulmonata. This grouping is defined by a ground pattern of two pairs of book lungs [1, 2]. The majority of recent analyses—molecular [3] and morphological [1]—suggest whip spiders are members of the Pedipalpi clade (Amblypygi, Uropygi and Schizomida), although there has been historical discussion; see e.g. Shultz [4]. Due to their highly modified sensory first pair of legs, whip spiders have to walk hexapodally using legs II–IV. They also possess a characteristic flattened body, allowing the animals to crawl into narrow spaces under rocks or tree bark, and they have spined, subchelate, raptorial pedipalps to grasp and immobilise prey. Today, the group has a tropical to subtropical distribution, with around 190 extant species in five families. A detailed overview of their biology and systematics can be found in Weygoldt [5]. A full species catalogue was offered by Harvey ([6]; updated online as [7]) and further published species counts can be found in Prendini [8].

### Fossil whip spiders

**Fig. 2:**
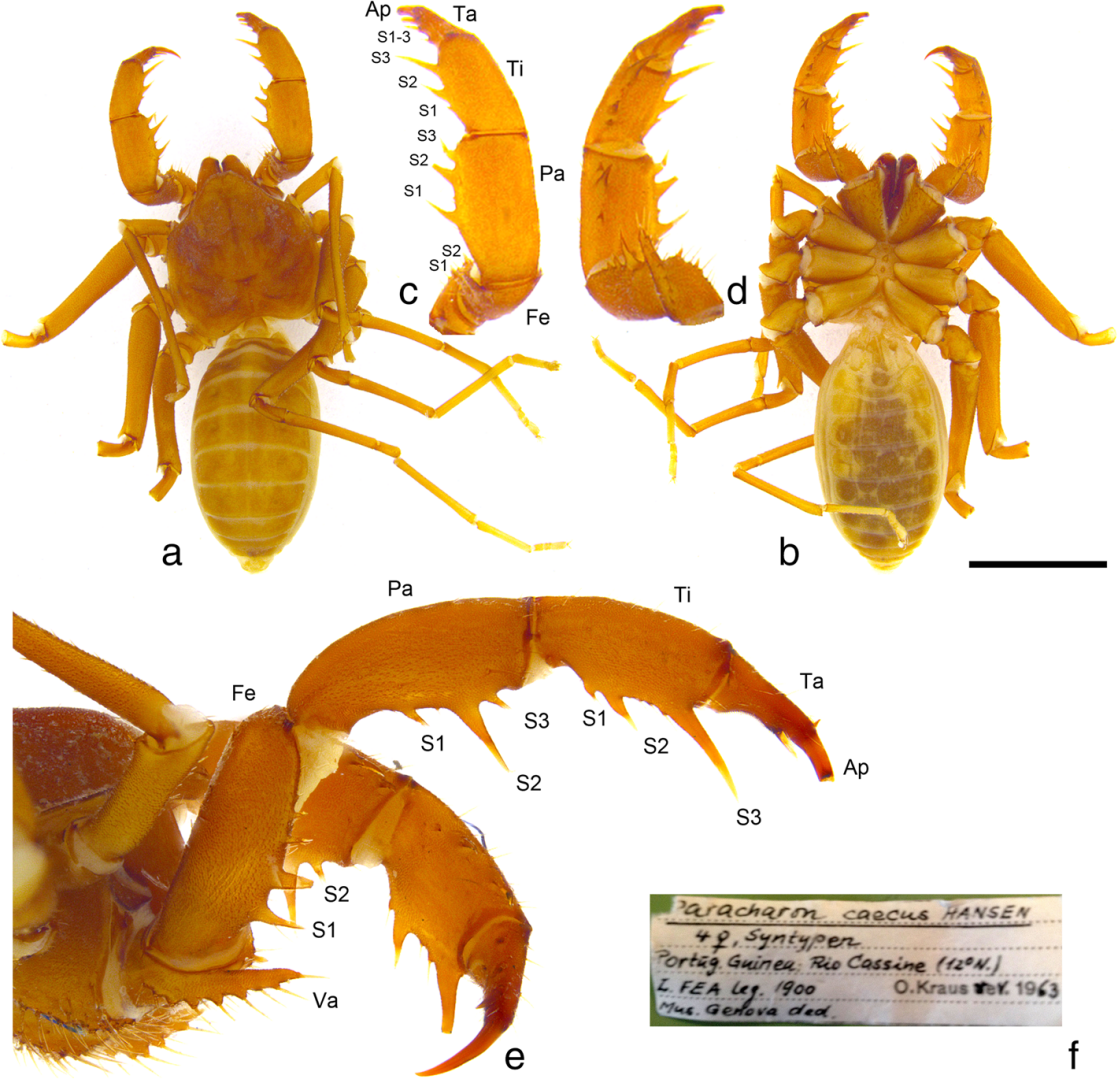
Images of a syntype of the only living paleoamblypygid *Paracharon caecus* Hansen, 1921 (Paracharontidae) which lives in termites nests in Guinea Bissau (West Africa). **a**-**b** Entire animal in dorsal and ventral view; scale bar equals 5 mm. **c**-**d** Close up of the pedipalp and its spination in dorsal and ventral view. **e** Enlarged lateral view of pedipalp in life position. **f** Original label. Abbreviations as in Fig. 1. Note the projecting anterior region of the carapace and the pedipalps which articulate up and down and have relatively weak spination. In carapace shape and details of the pedipalp this putatively plesiomorphic Recent species resembles the Carboniferous fossil *Graeophonus anglicus* Pocock, 1911

Whip spiders are rare as fossils. The oldest potential evidence for this group comprises some Middle Devonian (Givetian: ca. 390 Ma) cuticle fragments named *Ecchosis pulchribothrium* Selden & Shear, 1991 which have a trichobothrium on the patella [9]—a character otherwise only seen in living Amblypygi today. Unequivocal Palaeozoic members of the clade include five species from the Late Carboniferous Coal Measures (ca. 315–305 Ma) of Europe and North America. Scudder [10] described an isolated opisthosoma from Cape Breton in Nova Scotia as *Libellula carbonaria* Scudder, 1876 (his Figure 2, referred to as Fig. 1 in the text) and interpreted it as a dragonfly larva. Based on the discovery of a better preserved specimen from Mazon Creek in Illinois ([11]; see also [12]). Scudder later reinterpreted this find as a whip spider and created a new genus *Graeophonus* Scudder, 1890 to accommodate it as *Graeophonus carbonarius*. In a widely overlooked move, Pocock [13] did not feel that Scudder’s two North American specimens were conspecific and renamed the younger one *Graeophonus scudderi* Pocock, 1911. In the same monograph focussing on the British Middle Coal Measures Pocock [13] described *Graeophonus anglicus* Pocock, 1911 which—as the best preserved species—is the focus of the present study. The species is known from several well-preserved specimens [14]. Dunlop [15] described a Carboniferous whip spider from the Writhlington Geological Nature Reserve known only from its ventral surface. A full overview of historical work on Palaeozoic taxa is provided by Dunlop et al. [14].

The next amblypygids found in the fossil record are Cretaceous in age and come from the ca. 115 Ma Crato Formation of Brazil—Dunlop, & Martill [16] described a species known from a ventral prosoma and limbs, including distinctive amblypygid pedipalps, and Dunlop & Barov [17] augmented this with further details of the sternal region and details of the walking limbs. These confirmed its referral to the modern family Phrynidae. Engel & Grimaldi [18] documented a further Cretaceous species, *Kronocharon prendinii* Engel & Grimaldi, 2014 from the ca. 99 Ma Burmese amber from Myanmar, with high levels of morphological detail preserved. In the same publication these authors described a whip spider from the Eocene (ca. 52 Ma) Cambay amber of India—*Paracharonopsis cambayensis* Engel & Grimaldi, 2014, which has a similarly high fidelity of preservation. The youngest records are from Miocene (ca. 16 Ma) Dominican Republic amber [19, 20] and the probably contemporary Chiapas amber of Mexico [21, 22]. Petrunkevitch’s amber species was recently shown to be a *nomen dubium* [23], while at least the Dominican amber material is barely distinguishable from a modern species of *Phrynus* Lamarck, 1801 (Phrynidae) found in the Caribbean.

### Crown groups, stem groups and microtomography

When studying fossils a key question is when the oldest crown-group representatives of a given clade first appear (i.e. species descended from the most recent common ancestor of all extant members of that group). These data provide calibration points for molecular clock studies estimating dates of cladogenesis. Approaches vary, but time-calibrated phylogenies have traditionally been created using node-dating priors for relaxed clocks [24], and recent developments have shown that fossils can be coded directly into morphological matrices based on extant taxa, and used as calibration points—so-called total-evidence or tip-dating [25]. Coal Measures arachnids are particularly interesting for these purposes given both their great antiquity—more than 300 million years—and often surprisingly good preservation, particularly in siderite nodules. For example, recent molecular clock work on harvestman (Opiliones) has been successful in demonstrating that Carboniferous fossils which unequivocally resolve as members of two modern suborders (Eupnoi and Dyspnoi) lived alongside harvestmen with extinct body plans placed in a new suborder, Tetrophthalmi [26, 27].

Both these harvestman studies were aided by the application of X-ray microtomography: a technique that allows digital visualisation of the void left by the organism within the nodule. It is a methodology that has proved especially useful in revealing distal details of appendages, as well as ornamentation such as spines or tubercles, which are often buried deep in the host nodule, and are difficult to resolve using traditional methods of light microscopy [28–32].

For the present study, we selected the Carboniferous whip spider *Graeophonus anglicus* from the British Middle Coal Measures as a model organism. As previously noted, it was first described by Pocock [13] and was later placed by Petrunkevitch [33] in the living (derived, see below) family Phrynichidae. More recently, Weygoldt [5, 34] commented on the similarity between *G. anglicus* and the living whip spider species *Paracharon caecus* Hansen, 1921 (Paracharontidae), which is thought to have a number of plesiomorphic traits. Here, we offer the first photographs of type material belonging to this rare species (Fig. 2) for comparison with the fossils. This curious whip spider, found in West African termite nests, shows some similarities to the Coal Measures fossils in its carapace shape and the orientation and spination of its pedipalps. It was described as blind, although in the photograph there are hints of lateral eye spots on the right side of the dorsal shield (Fig. 2a). Based on a re-examination of Pocock’s fossils, Dunlop et al. [14] went further and explicitly referred *G. anglicus* to Paracharontidae, but could not resolve sufficient features to test this placement cladistically using the matrix of Weygoldt [34]. A principal aim of the present study was therefore to CT scan the fossils in the hope of yielding more details of the morphology of this important Carboniferous species—in particular from the appendages—and use this to explicitly test the phylogenetic position of *G. anglicus*. A primary objective was to demonstrate whether it is best considered a crown- or a stem-group whip spider. As part of the study, we also chose to include all well-preserved fossil amblypygids in the same cladistic analysis to assess the phylogeny and evolutionary history of the order.

## Methods

### Material

Three specimens of *Graeophonus anglicus*, were scanned from the Natural History Museum, London (NHMUK) collections: NHM In31234, NHM In31248, and NHM In31257. All originate from the Coseley Lagerstätte, a productive site from which specimens were collected in the late 1800s and early 1900s. The fossils—including these specimens—are typically preserved as three-dimensional voids within siderite concretions, sometimes with partial kaolinite infill. The deposit is Late Carboniferous (Bashkirian/Moscovian: Duckmantian) in age, or Westphalian B in traditional terminologies. The Duckmantian straddles the Bashkirian/Moscovian boundary, which Pointon et al. [35] place at around 315 Ma. Fossils were compared to Recent whip spiders in the collections of the Museum für Naturkunde Berlin. Comparative photographs of a syntype of the rare living species *Paracharon caecus* Hansen, 1921 (Fig. 2) were kindly provided by Jan Pedersen and Nikolaj Scharff from the Statens Naturhistoriske Museum, Københavns Universitet.

### Tomography

**Fig. 3:**
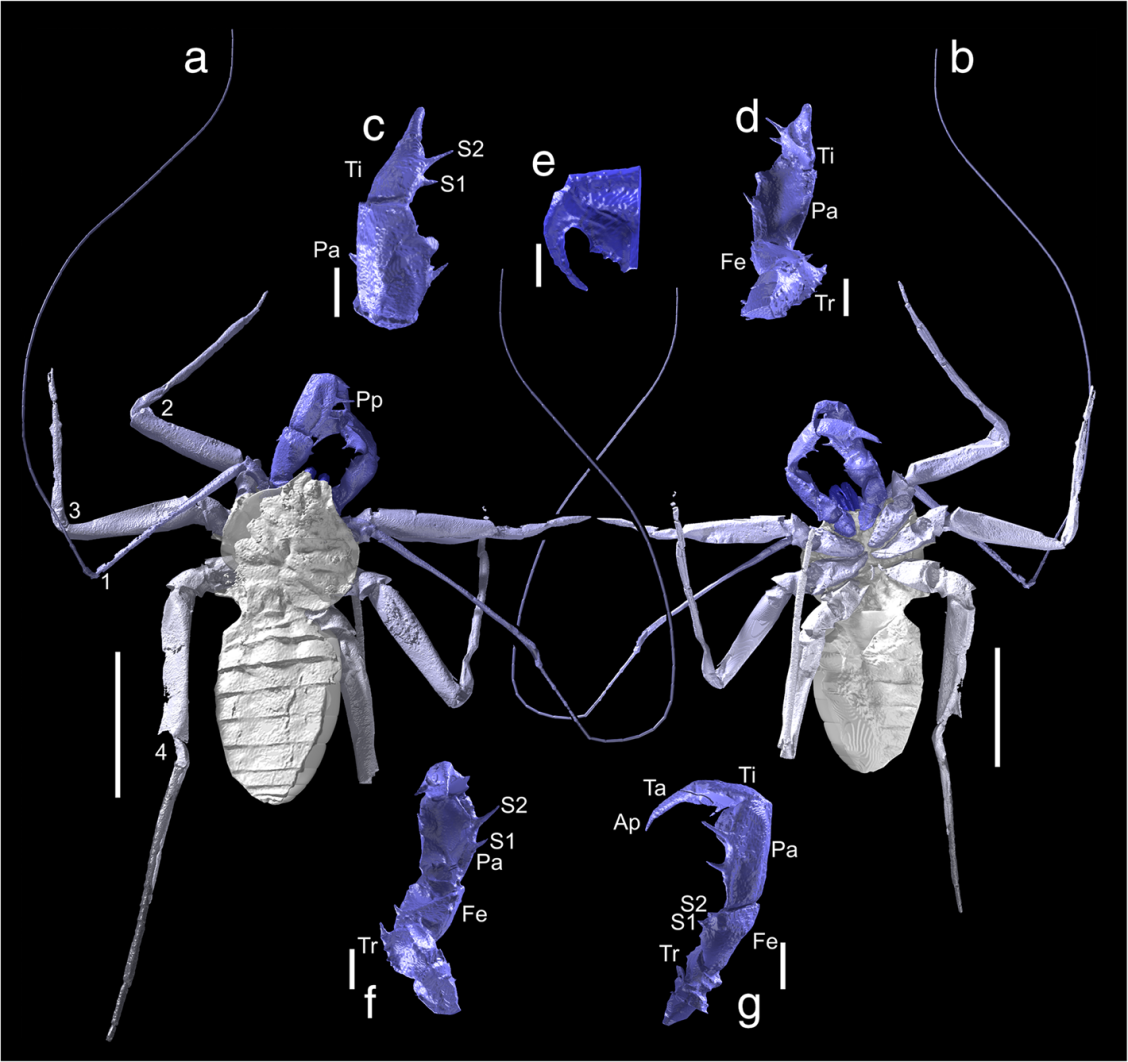
Tomographic reconstruction of *Graeophonus anglicus* Pocock, 1911; inferred/reconstructed anatomy partially transparent. **a**-**b** Whole animal in dorsal and ventral view; scale bar equals 5 mm. **c**-**d** Close up of the right pedipalp and its spination in dorsal and ventral view, tibial spination numbered; scale bar equals 1 mm. **e** A single chelicera, showing four teeth; scale bar equals 0.5 mm. **f**-**g** Close up of the left pedipalp and its spination in dorsal and ventral view, patellar spination numbered; scale bar equals 1 mm. Abbreviations as in Fig. 1

We scanned all specimens at the Natural History Museum, London, using a Nikon HMX-ST 225 and tungsten reflection target. Visualisations of the data (Fig. 3) allowed us to select one of the paratypes—NHM In31234—as the most promising specimen, a key criterion being well-preserved, three-dimensional pedipalps. During the scanning of this specimen, we collected 6284 projections at 195 kV/95 μA over 360 degrees rotation, with a 0.5 mm copper filter, and exposure of 1.4 s. We created volumes using CT Pro, and the 4MP (2000 × 2000) Perkin Elmer detector panel provided a voxel size of 15.8 μm. Digital visualisations were created using the SPIERS software suite, following the methods of Garwood et al. [36], and the model was rendered in the open source ray tracer Blender. We modelled missing elements of the anatomy in this program following the methods of Garwood and Dunlop [37] through comparison with living species, and then rendered them partially transparent in Fig. 3. Models are provided as SI to the current paper in the VAXML interchange format [38] (Additional file 1). We used this model as the basis of our redescription of the species; morphological terminology follows Weygoldt [5, 34], apart from the pedipalps where we use the scheme of Shultz [4].

### Cladistic Taxa and Characters Added

In order to assess the phylogeny of the fossil whip spiders we first re-ran the matrix of Weygoldt [34], but were unable to reproduce these results, recovering instead a polytomy with no reliable placement for fossil taxa. This is likely to result from differences in the search strategies and consensus tree calculations between Hennig86 and TNT, the latter used in the current study. Instead we chose to code fossil whip spiders into the matrix of Garwood et al [39], modified after Garwood & Dunlop [1], and based on the matrix of Pepato et al. [40], which in turn built on that of Giribet et al. [41]. In order to obtain internal resolution within the amblypygids, and to fully encompass their morphological disparity, we added numerous characters. The are fully described in the supplementary character statements (Additional file 2), and we summarise relevant character additions here. Character 38 reflects the number of cheliceral teeth within the amblypygids, which is either four or three depending on taxon (after Weygoldt [34]; character 1), and character 39 whether the distal-most tooth is bicuspate ([34]; character 2). Character 53 reflects the presence of a serrula; modified setae found on the cheliceral free finger in amblypygids and Schizomida. Character 54 further codes for whether the cheliceral serrula is more rounded—as in the whip spiders and the two Protoschizomidae genera of the schizomids—or toothlike as seen in the schizomid family Hubbardiidae (after Cokendolpher & Reddell [42]).

A key difference seen in the pedipalps of *Paracharon* and *Graeophonus*, in contrast with other whip spiders, is a more vertical plane of motion (Figs. 2 and 3), associated with a presumably plesiomorphic pediform state for these limbs. The modification in plane of motion is probably associated with prey capture, and has been added as character 62. Much of the internal resolution within Amblypygi is based on the arrangement and nature of the spines of the pedipalp, associated with the limbs’ raptorial nature. Character 64 reflects whether the pedipalps have a row of femoral spines ([34]; character 7), character 65 on whether the trochanter has a distinctive ventral apophysis, and character 66 on whether this is present as a spine ([34]; character 4). Character 69 has been modified to reflect whether palpal tarsus is subdivided or fused. This character is modified after Weygoldt ([34]; character 14); we highlight that our pedipalp tarsus is equivalent to Weygoldt’s distitarsus (the two parts of which are fused within the Neoamblypygi clade; see below), and distal part of this we term the apotele. Character 71 reflects the absence or presence of a dorsal row of patellar spines on the pedipalp which, in some groups, form a catching basket, and in others (e.g. Phrynichidae) is more of a prehensile structure (a distinction coded in character 75). This row often consists of three principal spines, with the distal-most largest, which is coded in character 72 ([34]; character 8). Whether these sequentially decrease in length towards the base (i.e. proximally) is coded as absent or present in character 73 ([34]; character 10), and the subsequent character, 74, records if the most proximal spine of the three principal ones is highly reduced ([34]; character 11). Character 76 codes for the presence of spines on the palpal tarsus.

We have additionally added a limb character to reflect the whip-like first limbs of whip spiders; character 83 is coded based on the number of subdivisions of the tibia of the first leg. This segment was chosen as it is more likely to be preserved in fossils, being more proximal than the (also subdivded) tarsus, and also on the basis of Weygoldt ([34]; character 18). We recognise five states: unmodified, 16 segments, up to 23, 25 segments, and more than 25 segments. Whilst this is largely only informative for the living species, it can also be coded for the amber fossils *Paracharonopsis* from the description of Engel & Grimaldi [18]. Whilst a rough figure for a *Kronocharon* species figured by Wunderlich [43] is obtainable, we have coded this as unknown for this species due to difficulty in differentiating the podomeres. We highlight that as part of ongoing development of the matrix we have added a number of further characters. These are listed in the character statements supplementary file (Additional file 2).

The previously published arachnid phylogeny of Garwood et al [39] included three whip spider genera; *Charinus*, *Musicodamon*, and *Paraphrynus*, as well as a generic coding for Amblypygi as a whole. In order to include a representative of every extant family—and to test the position of the extinct *Kronocharon* in more detail—we have added the genera *Charon, Paracharon* and *Stygophrynus.* The former were coded from Weygoldt ([5, 34]; character 18). We note that the new photos of the type specimen of *Paracharon*, suggest lateral eyespots are present (Fig. 2a). Hence we have coded lateral eyes as present herein, but the number of lenses and the nature of the rhabdomes as unknown. *Stygophrynus* [44] was coded on the suggestion of a reviewer, as it differs in some important aspects of the pedipalp from *Charon* even though both genera are currently placed in the same family. We also added the three known and reasonably complete fossil genera: *Graeophonus*, coded from the results herein and redescription of Dunlop et al. [14], and *Paracharonopsis* and *Kronocharon* from Engel & Grimaldi [18]. We omitted the three species described by Petrunkevitch [12] as we consider the species descriptions unreliable, and the species described by Schawaller [19, 20] which is essentially modern.

### Cladistic Analysis

We analysed this matrix using TNT v.1.1. ([45] ; made available with the sponsorship of the Willi Hennig Society), using a traditional search and unordered multistate characters. The data matrix is available as Additional file 3 in a TNT-file ready format. Our searches comprised tree bisection-reconnection [TBR] with 1000 replicates, saving 100 trees per cycle. These were carried out under equal and implied weights. For all analyses, TNT was used to create a strict consensus tree which was exported as an SVG into Inkscape. For our implied weights analyses, we present the strict consensus of an arachnid-wide analysis at a *k* value of 3, and for the amblypygids, an additional strict consensus of all tree topologies recovered from 88 *k* values ranging from 0.001 to 122.0. We further explored the matrix by running the analyses with differing taxa and characters excluded to explore their impact. Resampling of all analyses was conducted within TNT. For our equal weight analysis, we provide jackknife ([46]; 33% removal probability, 10,000 replicates), bootstrap ([47] ; 10,000 replicates) and Bremer support [48]. For the implied weights analysis, *k* =3, supports are included through symmetric resampling ([49] ; change probability 33%, 10,000 replicates). Where applicable, nodal support values are shown as absolute frequencies.

## Results

### Morphological interpretation

**Fig. 4:**
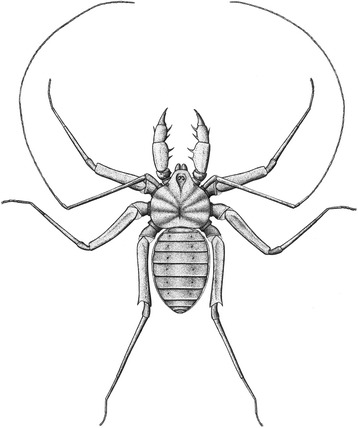
*Graeophonus anglicus* as it may have appeared in life; distal parts of leg I hypothetical, but based on comparisons with extant material



Much of the basic somatic morphology in *Graeophonus anglicus* was covered by Pocock [13], Petrunkevitch [50] and Dunlop et al. [14]. In brief, the prosomal dorsal shield (or carapace) is a single plate, somewhat reniform in outline, but with a distinct projection of the anterior median region (Figs. 3a and 4, Additional file 1; movie included as additional file 4) similar to the condition in *Paracharon caecus* (Fig. 2a), as elucidated by Weygoldt ([34], character 28). The centre of the dorsal shield in *G. anglicus* has a deep depression (the fovea) which probably acted as an attachment site for the muscles of the sucking stomach. Several grooves radiate out from this depression. Previous interpretations picked up the median eye tubercle in *G. anglicus*, and the CT data confirmed the presence of lateral eye tubercles (Fig. 3a) which were considered equivocal in previous studies.

**Additional file 4:** A video showing the tomographic reconstruction of *Graeophonus anglicus* Pocock, 1911 presented herein; inferred/reconstructed anatomy partially transparent. (AVI 87.2 mb)


The chelicerae of *Graeophonus anglicus* were also largely equivocal in previous studies, but the CT data reveals them as two-segmented, clasp-knife structures similar to those of living species (Fig. 3e). In modern whip spiders the fang opposes a series of internal teeth (Fig. 1), whose number and structure can be phylogenetically informative ([34]; character 13). The CT scan was able to resolve the presence of four internal teeth in *G. anglicus*, although the most proximal one may not be preserved at its full length. Also of significance is the fact that, as in *Paracharon*, the uppermost or distal tooth has only one cusp. In all other living whip spiders the upper tooth has two cusps. The coxo-sternal region is well-preserved and for the first time the CT scan picks up the small coxae of leg I (Fig. 3a). The coxae of legs II-IV are very clearly preserved and coxa II in particular is quite tuberculate (Fig. 3b).

Pedipalp morphology is a key character for resolving whip spider relationships. Previous studies [14] recognised two dorsal spines on the femur of the pedipalp in *Graeophonus anglicus*. This is again similar to the condition in *Paracharon caecus* (Fig. 2c) and was used as evidence for referring the Carboniferous fossil to Paracharontidae. The CT scans allowed us to investigate pedipalp morphology in more detail (Fig. 3c, d, f, g). We could confirm the general observation that the pedipalps in *G. anglicus* primarily articulate up and down in a vertical plane (Fig. 3b). This is more like the condition in *P. caecus* (Fig. 2e); in more derived whip spiders the pedipalps primarily articulate from side to side.

In detail, we could resolve that the trochanter of the pedipalp has a ventral apophysis (Fig. 3d,f)—a character for all Amblypygi—and that in *Graeophonus anglicus* it is more like a flange and not reduced to a spine ([34]; character 4). We could confirm that, like *Paracharon caecus* (Fig. 2c), the femur of the pedipalp in *G. anglicus* lacks a prominent row of dorsal spines (Weygoldt [34], character 7) and has only two short spines here (Fig. 3g). Weygoldt [34], like many authors, referred to the next pedipalp article in whip spiders as the tibia, however we follow Shultz [4] and recognise a more conventional series of articles for an arachnid pedipalp: namely a femur, patella, tibia, tarsus and apotele. In this scheme, we can argue that *G. anglicus* has two patellar spines (Fig. 3f). It also has, like *P. caecus* (Fig. 2c), only two prominent tibial spines (Fig. 3c). However, in the photograph of *P. caecus* (Fig. 2c) there is a small (proximal) tibial spine which was not shown in Weygoldt’s drawings. Three prominent tibial spines are seen in more derived living species ([34]; character 8). The distal tip of the pedipalp in *G. anglicus* appears to be offset against the preceding element forming a pretarsus or apotele (Fig. 3g). In Weygoldt’s [34] character 14 terminology this would be referred to as a “divided distitarsus”. We prefer to interpret and score it as a tarsus which is (still) separate from its apotele. In more derived whip spiders (namely the Unidistitarsata; see below) the tarsus and apotele are fused.

As in living whip spiders, the first leg of *Graeophonus anglicus* appears to be long and slender. The CT scan can trace leg I down to the first few articles of what is clearly a subdivided tibia (Fig. 3a,b, Additional file 1). This implies that leg I was antenniform as in Recent taxa, but the exact number of tibial segments ([34]; character 18) remains equivocal. Legs II-IV of *G. anglicus* are more complete, and III and IV can now be traced down to near their tips (Fig. 3a-b). Some legs show evidence for a pair of tarsal claws, but the presence or absence of a pad-like pulvillus between these claws in *G. anglicus* is equivocal. The femur is quite robust and, at least in legs II and III, it is preserved with the prolateral surface uppermost. This may well reflect its orientation in life since modern whip spiders have a similar leg configuration to help them to crawl into narrow spaces. The patella is small and bell shaped. The tibia of leg IV is divided into a basi- and distitibia as expected, and the distitibia itself appears to be undivided ([34]; character 23). The opisthosoma is oval with a series of tergites (Figs. 3a-b and 4) which are shorter towards the anterior and posterior ends. Ventral sacs on the underside of the opisthosoma are equivocal. A reconstruction of the likely appearance in life is presented in Fig. 4.

### Cladistic results and discussion

Traditionally, whip spiders were broadly divided into so-called pulvillate and apulvillate taxa (e.g. [51]) based on the presence or absence of this small fleshy pad called the pulvillus at the ends of legs II-IV. Weygoldt [34] tested this hypothesis cladistically and recovered (Paracharontidae (Charinidae (Charontidae (Phrynichidae + Phrynidae)))), rendering the ‘pulvillate’ taxa paraphyletic. As an alternative way of expressing this, he recognised two suborders: Paleoamblypygi (for Paracharontidae) and Euamblypygi for the other four families. The euamblypygids were further subdivided into the infraorders Charinidae (for the family of the same name) and Neoamblypygi. Finally, neoamblypygids were divided into the superfamilies Charontoidea (for Charontidae) and Phrynoidea (for Phrynichidae and Phrynidae). Note that the phrynoids are the most derived group in this scheme, and equivalent to the ‘apullvillates’ in Quintero’s earlier scheme. Weygoldt’s model was largely adopted in subsequent classifications, such as the catalogue of Harvey [6], albeit with the modification of the infraorder name Charinidae to Charinina in Prendini [8]. Within the neoamblypygids, the recent study of amber fossils by Engel & Grimaldi [18] introduced another clade name, Unidistitarsata, which encompassed their extinct genus *Kronocharon* Engel & Grimaldi, 2014 + Phrynoidea (but see below).

### Amblypygi

**Fig. 5:**
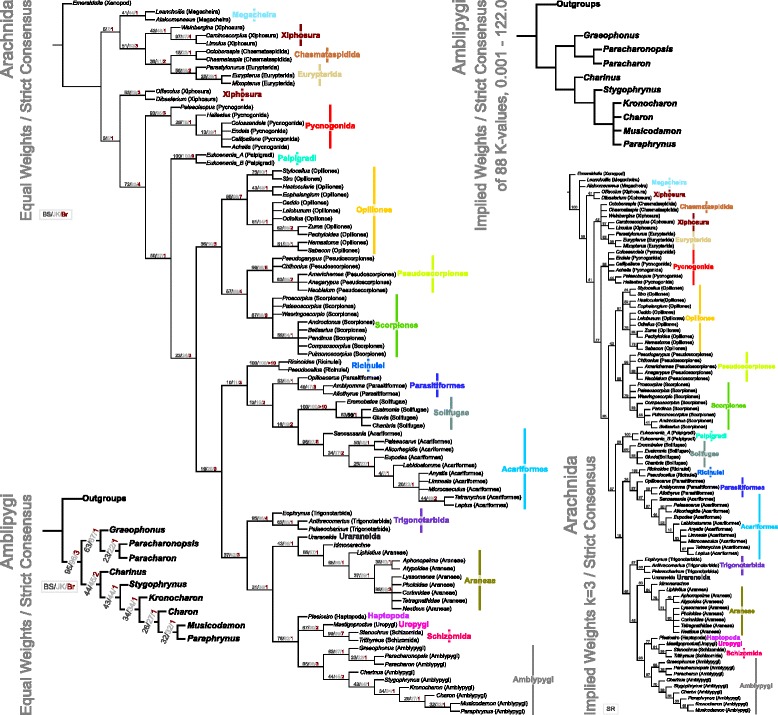
The results of the cladistic analysis of chelicerates and arachnids used to place fossil amblypygids. Shown is the strict consensus tree run under an equal weighting scheme, bootstrap, jackknife, and Bremer support values, a strict consensus tree recovered using implied weighting (*k* = 3), and a strict consensus of the trees recovered under 88 implied weights analyses at varying *k* values

**Fig. 6:**
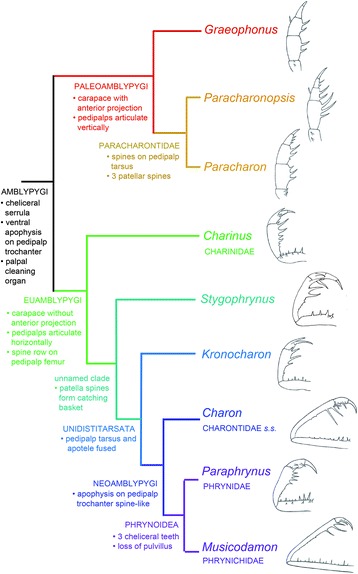
Results of the cladistic analysis highlighting key character transformations, together with sketch reconstructions of the pedipalps of our selected terminal taxa (not to scale). Note the changes in pedipalp orientation, structure and spination going up through the phylogenetic tree

**Fig. 7:**
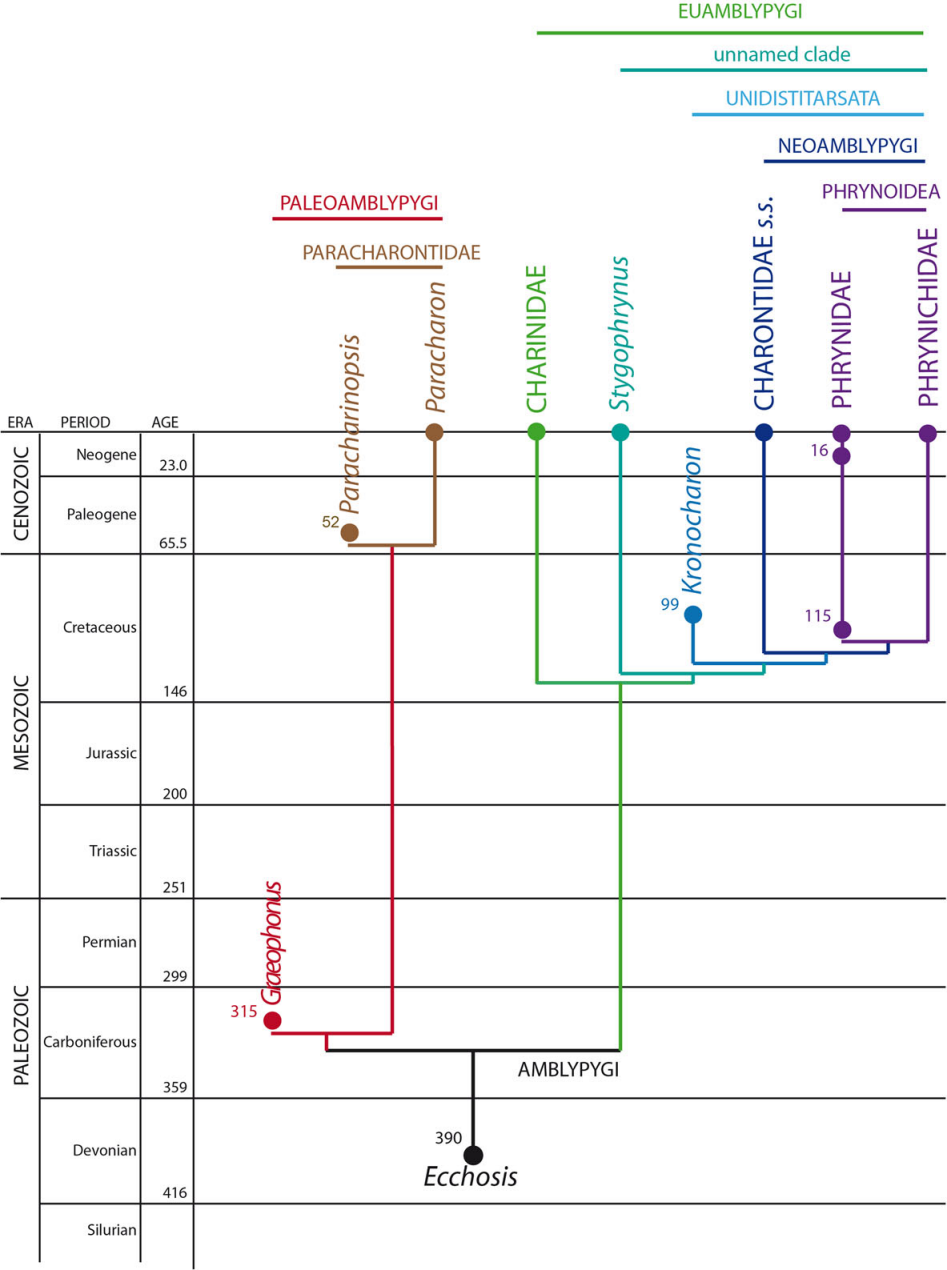
The recovered phylogeny expressed as the current higher taxon/family structure and superimposed on the the known fossil record. Circles indicate fossil occurrences with their approximate dates in millions of years. The enigmatic *Ecchosis* is tentatively treated as being close to whip spider origins. Amblypygi and Palaeoamblypgyi can be dated from the Carboniferous to Recent; Paracharontidae from the Eocene to Recent. Euamblypygi can be dated from the Cretaceous to Recent, while the phrynid from the ca. 115 Ma Crato Formation of Brazil implies that the remaining lineages (Unidistitarsata, Neoamblypygi, Phrynoidea) were also present during the late Cretaceous

Cladistic analysis of our matrix using traditional search options (TBR) and equal weights (EW) resulted in 96 trees of 520 steps. Implied weights analysis (*k* = 3), resulted in 24 trees of 41.38 steps. We present these trees in Fig. 5. Equal weights analysis recovers Amblypygi in a polytomy with Uropygi and Haptopoda, and ingroup whip spider relationships of the form (*Graeophonus (Paracharonopsis* + *Paracharon*)) + (*Charinus* (*Stygophrynus* ((*Kronocharon* (*Charon* (*Musicodamon* + *Paraphrynus*))))). The basic phylogenetic structure is thus largely in accordance with the results of Weygoldt [34]. We also present our results from this analysis mapped onto the main character transformations and the pedipalp structure of our terminal taxa (Fig. 6), and plotted against geological time (Fig. 7). Implied weights analyses recovers ((Amblypygi) (Haptopoda (Uropygi + Schizomida)), but shows some internal instability within the amblypygids. This results from the fossil taxon *Kronocharon*, which has a placement matching that of equal weights at high *k* values, but at lower ones is resolved as sister group to *Musicodamon*. Other relationships are unchanged. We note that discussions regarding the merits of different weighting schemes, and parsimony in comparison to probabilistic methods are ongoing [52, 53]. In this work we opt to focus our discussion on the equally weighted analysis, recognising this is potentially a more accurate form of parsimony analysis than implied weights, but could be overly-precise.

Looking at the character distribution on this tree in detail, Amblypygi is defined in our analysis by six apomorphies, although not all of them can be seen in all the fossils. These are: (1) the presence of serrula on the chelicerae, (2) a ventral apophysis on the pedipalp trochanter, (3) presence of a palpal cleaning organ, (4) a dorsal row of patellar spines on the pedipalp, (5) presence of a pulvillus on legs II-IV, albeit with a reversal in the most derived genera, and (6) the presence of ventral sacs. Note that serrula, a pulvillus and ventral sacs are not unique for Amblypygi and can also occur in some other arachnid orders.

### Paleoamblypygi

The analysis supports a monophyletic suborder Paleoamblypygi *sensu* Weygoldt [34], consisting of *Graeophonus*, *Paracharon* and *Paracharonopsis* (Fig. 5)*.* A single putative apomorphy supports Paleoamblypygi in our analysis, namely the anterior projection at the front of the prosomal dorsal shield. This is not seen in the Euamblypygi (see below), in which the anterior margin is straight or only slightly rounded. Were this carapace projection to be interpreted as a plesiomorphic character, we must entertain the possibility that the paleoamblypygids are a grade rather than a clade, but this was not the most parsimonious result in the present analysis. Outgroup comparison does suggest that other paleoamblypygid features like the vertically articulating pedipalps are probably plesiomorphic (as it is for other Pedipalpi).

In contrast to Dunlop et al. [14], we now formally exclude *Graeophonus* from Paracharontidae (see Systematic Palaeontology) and treat it as a stem-paleoamblypygid. Our justification for this is that *Graeophonus anglicus* has a much simpler pattern of pedipalp spines compared to other whip spiders. Characters relating to this spination explicitly group *Paracharon* closer to *Paracharonopsis* (see below). This result is similar to Tetlie & Dunlop’s [54] conclusions about the Coal Measures whip scorpions. The earliest fossil whip scorpions lack projecting apophyses on the pedipalp. These define the (Cretaceous-Recent) whip scorpion family Thelyphonidae as a crown group which can be defined by more raptorial—and presumably more efficient—pedipalp used for prey capture. Likewise, the palp of *Graeophonus* suggests that whip spiders also seem to show a trend in which the pedipalps become increasingly better adapted for restraining their prey (Fig. 6).

With respect to the other Coal Measures whip spiders, the original figures of *Sorellophrynus carbonarius* (Petrunkevitch, 1913) from Mazon Creek suggest that this genus also has the paleoamblypygid character of a carapace projection. The status of *Thelyphrynus elongatus* Petrunkevitch, 1913, also from Mazon Creek, is harder to assess. The carapace is figured as more rounded anteriorly, but the original figures imply that the carapace and pedipalp are not well-preserved. In lieu of a formal redescription we provisionally place this genus as a paleoamblypygid too.

### Paracharontidae

The family Paracharontidae is restricted here to *Paracharon* and *Paracharonopsis* (Fig. 6)*.* It is defined in our dataset by the presence of several small spines on the pedipalp tarsus (Fig. 2c), a character which is—convergently in our analysis—present in *Charinus* too. This character is reversed in the more derived whip spiders in which the tarsus and apotele also fuse into a single (and spineless) tip. More generally, we suggest that the pedipalps of the amber and living palaeoamblypyid genera are more heavily spined than the pedipalps of *Graeophonus anglicus*. For example the patella bears three spines in Paracharontidae, but only two in *G. anglicus* (Figs. 3 and 6).

### Euamblypygi

Weygoldt’s [34] suborder Euamblypygi—i.e. (*Charinus* (*Stygophrynus* ((*Kronocharon* (*Charon* (*Musicodamon* + *Paraphrynus*))))) among our terminal taxa—is defined in our dataset by (1) pedipalps which articulate primarily in a horizontal plane and (2) a row of dorsal spines on the pedipalp femur, rather than just a couple of isolated spines as in Paleoamblypygi; see also Fig. 6. The biology of living *Paracharon caecus* is unknown but we suspect that both this species, and the fossil paleoamblypygids, grab down on prey items immediately in front of them. Almost all living whip spiders are euamblypygids. We presume that the horizontal, side-to-side, action of their pedipalps conferred a considerable evolutionary advantage, enabling broad sweeping movements to capture prey which is further away. The elongation of the pedipalps in derived euamblypygids supports this general hypothesis.

### Unnamed clade

The next clade recovered in our analysis is (*Stygophrynus* (*Kronocharon* (*Charon* (*Musicodamon* + *Paraphrynus*)))). The principal apomorphy which supports this group is the tendency for the distal ventral patellar spines on the pedipalp to form a ‘catching basket’ (subsequently modified into the phrynichid ‘hand’). It is also supported by a longer number of tibial elements in the first pair of legs and the loss of the coxal gland opening on the segment bearing the third pair of legs. Traditionally *Stygophrynus* and *Charon* were placed together as the family Charontidae. Our results challenge the monophyly of this family (see also below) especially given the fact that *Stygophrynus* retains a separate tarsus and apotele in the pedipalp while *Charon* has the more derived character of a fusion of these elements. In our analysis the two extant genera are also split apart from each other by the extinct *Kronocharon* which also has a fused pedipalp tip (Fig. 6; although this does move up-tree in some implied weights analyses). To accommodate this result we could assign *Kronocharon* to Charontidae too, but this would still leave the family paraphyletic and containing genera having quite different pedipalp architectures. Since we were not comprehensive in covering extant genera we prefer for now to place *Stygophrynus* as the sister group to the Unidistitarsata (see below) and restrict Charontidae to the genus *Charon* as Charontidae *sensu stricto*. We concede that it may prove necessary to recognise a separate family for *Stygophrynus*, but we are reluctant to add a monogeneric family to the nomenclature at this stage.

### Unidistitarsata

This clade is defined in our dataset by the key character of the fusion of the tarsus and apotele into a single element. In our analysis the Burmese amber genus *Kronocharon* resolved as sister group to the Neoamblypygi (but see also implied weights analysis). This position for *Kronocharon* differs from the original interpretation of Engel & Grimaldi [18], who proposed that the fossil genus was the sister group of the Phrynoidea (i.e. *Musicodamon* + *Paraphrynus*), forming the Unidistitarsata based on the diagnostic apomorphy of an undivided pedipalp tip (Fig. 6). However, we contend that—contra Engel & Grimaldi [18]—*Charon* also has, like phrynoids, an undivided distitarsus, as shown in Figure 15 of Weygoldt [34], and reflected in the scoring of his character 14 (see also [55] for example). As noted above *Stygophrynus*, by contrast, retains a pedipalp tip which is divided. The most derived whip spiders also have the greatest number of elements in the leg I tibia. It may be possible to score the number of tibial elements in leg I for *Kronocharon* based on Wunderlich [43], who reported 65 elements beyond the patella. However, since the exact number of tibial as opposed to tarsal elements is not given here we have chosen to treat this character in the amber genus as equivocal.

### Neoamblypygi

The neoamblypgids, (C*haron* (*Musicodamon* + *Paraphrynus*)) from our selected terminal taxa, are defined in this analysis by the apomorphy of a spine-like ventral apophysis on the trochanter of the pedipalp. By contrast in *Kronocharon* this apophysis is described as being a large and carina-like [18], rather than explicitly a spine. As noted above, in our scheme Charontidae is no longer monophyletic, and we recognise Charontidae *sensu stricto* (consisting of *Charon* only) as the sister-group to Phrynoidea.

### Phyrinoidea

Finally the two most derived genera in our dataset (*Musicodamon* and *Paraphrynus*) belong to the Phrynoidea (or Phrynida), which is defined here by (1) the reduction of four cheliceral teeth to three and (2) the loss of the pulvillus. Indeed, as noted above, an older name for the same clade in the literature is Apullvillata.

## Systematic palaeontology

Order AMBLYPYGI Thorell, 1883

- Suborder PALEOAMBLYPYGI Weygoldt, 1996
- Stem-PALEOAMBLYPYGI

### Included genera

†*Graeophonus* Scudder, 1890; †*Sorellophrynus* Harvey, 2002; †?*Thelyphrynus* Petrunkevitch. 1913.

- Genus †*Graeophonus* Scudder, 1890
- †*Graeophonus anglicus* Pocock, 1911

### Material

NHM 31234 (paratype). From Coseley, near Dudley, Staffordshire UK. British Middle Coal Measures, Late Carboniferous (Duckmantian) (Figs. 3 and 4, Additional file 1).

### Description

Description as Dunlop et al. [14]: here we primarily focus on novel morphological features revealed through microtomography (Fig. 3). Total length 11.5 mm. Prosomal dorsal shield reniform, but with wide anterior projection; shield length 4.6 mm, maximum width 5.3 mm. Dorsal shield with median longitudinal depression (the fovea) and several depressions radiating out from this structure. Pair of median eyes on the anterior projection, borne on a tear-drop shaped tubercle; small lateral eye tubercles in an anterolateral position can also be resolved; number of individual lenses equivocal.

Chelicerae small, of the ‘clasp-knife type, projecting forwards beyond the anterior margin prosomal shield and composed of two articles (Fig. 3e): a basal element (or paturon; min. 8 mm long) opposed by a gently curving and tapering distal fang (1 mm in length). Basal element bears four teeth, the most distal of which has only one cusp. Pedipalps robust and bearing numerous spines (Fig. 3c,d,f,g); total length ~7 mm. Trochanter with flange-like ventral apophysis. Femur broadens distally and bears only two small dorsal spines. Patella broad, slightly procurved on the mesal margin and here bearing two spines, the proximal one slightly shorter (0.3 mm) than the distal one (0.6 mm). The same article has a further distal spine on the outer surface (0.4 mm). Tibia with two mesal spines, proximal shorter (0.3 mm) than distal (0.6 mm), and a prominent outer distal spine. Tarsus separate from terminal apotele; both without spines.

Legs gracile, leg I antenniform. Leg I coxa small, triangular. Leg I trochanter more than twice as wide as long. Leg 1 femur slender, narrowing slightly distally. Leg 1 patella small, bell shaped. Leg 1 tibia incomplete but was evidently subdivided; more distal elements equivocal but overall habitus implies an antenniform appendage. Legs II-IV more robust. Coxae subtriangular; trochanters bell-shaped. Femora somewhat flattened, apparently with thin margins and becoming slightly narrower distally. Femur IV with hook-like ventral projection at its distal margin. Patellae short and bell shaped. Tibia slender and divided into a basi- and distitibia; these elements are not further subdivided. Basitarsus undivided, but tarsus divided into three tarsomeres, with terminal claws visible in leg III. Pulvillus equivocal. Tritosternum or additional expected sternal elements between the leg coxae equivocal.

Opisthosoma oval, flattened; length 7 mm, maximum width 4 mm. First tergite short, next six longer and approximately of equal length, posteriormost four visible tergites increasingly shorter. Ventral sternite pattern largely matches that of the corresponding tergites. Ventral sacs on underside of the opisthosoma equivocal.

- Family PARACHARONTIDAE Weygoldt, 1996

### Included genera

*Paracharon* Hansen, 1921; †*Paracharonopsis* Engel & Grimaldi, 2014.

- Suborder EUAMBLYPYGI Weygoldt, 1996
- Family CHARINIDAE Quintero, 1986

### Included genera

*Catageus* Thorell, 1899; *Charinus* Simon, 1892; *Sarax* Simon, 1892.

- Unnamed clade

### Included genus

*Stygophrynus* Kraepelin, 1895.

- Clade UNIDISTITARSATA Engel & Grimaldi, 2014

### Included genus

†*Kronocharon* Engel & Grimaldi, 2014.

- Infraorder NEOAMBLYPGI Weygoldt, 1996
- Superfamily CHARONTOIDEA Simon, 1892
- Family CHARONTIDAE Simon, 1892 *sensu stricto*

### Included genera

*Charon* Karsch, 1879.

- Superfamily PHRYNOIDEA Blanchard, 1852
- Family PHRYNIDAE Blanchard, 1852

### Included genera

*Acanthophrynus* Kraepelin 1899; †*Britopygus* Dunlop & Martill, 2002; *Heterophrynus* Pocock, 1894; *Paraphrynus* Moreno, 1940; *Phrynus* Lamarck, 1801.

- Family PHRYNICHIDAE Simon, 1892

### Included genera

*Damon* C.L. Koch, 1850; *Euphrynichus* Weygoldt 1995; *Musicodamon* Fage, 1839; *Phrynichodamon*, Weygoldt 1996; *Phrynichus* Karsch, 1879; *Trichodamon* Mello-Leitao, 1935; *Xerophrynus* Weygoldt, 1996.

## Conclusions

Tomographic investigation of the Carboniferous amblypygid *Graeophonus anglicus* reveals the least modified pedipalps of any whip spider, living or extinct. These appendages still primarily articulate up and down, in common with typical arachnid walking legs. They have relatively few spines to facilitate prey capture: namely two small dorsal spines on the femur, two larger spines on the patella and three (two mesal, one lateral) on the tibia. *G. anglicus* is placed in the Carboniferous–Recent suborder Paleoamblypygi (Fig. 7), which can be defined based on the projecting anterior part of the carapace (Fig. 6). However, the fossil species can be excluded from the Eocene–Recent group Paracharontidae which has more pedipalp spines; specifically three patella spines and additional small tarsal spines. Most whip spiders belong to the Cretaceous–Recent suborder Euamblypygi (Fig. 7), in which the pedipalps primarily articulate from side to side, i.e. with a horizontal plane of motion. The Cretaceous phrynid from the Crato Formation of Brazil implies that all three euamblypyid families should have been present in the Cretaceous (Fig. 7). The relationships we recover (Fig. 5) largely support the 1996 phylogeny of Weygoldt, although the recently described Burmese amber genus *Kronocharon* is placed a node deeper in the tree compared to its original interpretation. This analysis demonstrates that whip spider phylogeny is effectively reflected in the evolution and modification of the group’s pedipalps (Fig. 6). Trends which can be recognised include (1) fusion of the tarsus and apotele to define the Unidistitarsata, (2) appearance of a spine-like ventral apophysis on the pedipalp trochanter, and a tendency to concentrate the patella spines distally into a ‘catching basket’ which define the Neoamblypygi, and (3) loss of the pulvillus on the legs and reduction in the number of cheliceral teeth defining the Phrynoidea. Several of the most derived phrynoid genera also have extremely long pedipalps, in which the distally highly concentrated patellar spines form the so-called phrynichid ‘hand’. These results provide a framework for testing the position of future fossil discoveries.

## Additional files

Additional file 1: File format:.vaxml (see [38]). Title: Tomographic reconstruction of *Graeophonus anglicus* Pocock, 1911. Description: A 3D mesh model of *Graeophonus anglicus* in the VAXML interchange format. (ZIP 45.3 mb)

Additional file 2: File format:.pdf. Title: Character statements. Description: Morphological characters statements for the characters used in the current analysis. (PDF 507 kb)

Additional file 3: File format:.tnt (see [45]). Title: Cladistic matrix. Description: Cladistic matrix used in the current analysis, in a TNT ready format. (TNT 44 kb)
